# Prevention and control of HPV and HPV-related cancers in France: the evolving landscape and the way forward – a meeting report

**DOI:** 10.1186/s12919-023-00271-0

**Published:** 2023-08-03

**Authors:** Dur-e-Nayab Waheed, Catherine Weil Olivier, Didier Riethmuller, Eduardo L. Franco, Jean Luc Prétet, Marc Baay, Nubia Munoz, Alex Vorsters

**Affiliations:** 1 Centre for Evaluation of Vaccination, Vaccine and Infectious Disease Institute University of Antwerp, Antwerp, Belgium; 2 Retired, Neuilly-Sur-Seine, France; 3 Department of Obstetrics and Gynecology, University Medical Center of Grenoble, Grenoble, France; 4 Division of Cancer Epidemiology, McGill University, Montreal, Canada; 5 Papillomavirus National Reference Center CHU, Besançon, France; 6 EA3181 Université Bourgogne Franche-Comté, Besançon, France; 7 P95, Epidemiology and Pharmacovigilance Consulting and Services, Leuven, Belgium; 8 National Cancer Institute, Bogotá, Colombia

**Keywords:** Human papillomavirus, HPV, Vaccination, Cervical cancer, Prevention, Screening, France

## Abstract

Misinformation regarding HPV vaccine safety and benefits has resulted in low coverage within the eligible French population. HPV vaccination is safe and efficacious in preventing HPV infections in adolescents. However, reaching optimal coverage in countries such as France is challenging due to misinformation, among other factors. Moreover, disparities exist in cervical cancer screening programs. To support the government health promotion policy aimed at improving prevention and control of HPV-related cancers in France, the Human Papillomavirus Prevention and Control Board (HPV-PCB), in collaboration with local experts, held a meeting in Annecy, France (December 2021).

HPV-PCB is an independent, multidisciplinary board of international experts that disseminates relevant information on HPV to a broad array of stakeholders and provides guidance on strategic, technical and policy issues in the implementation of HPV control programs.

After a one-and-a-half-day meeting, participants concluded that multi-pronged strategies are required to expand vaccination coverage and screening. Vaccine acceptance could be improved by: 1) strenghtening existing trust in clinicians by continuous training of current and upcoming/pre-service healthcare professionals (HCPs), 2) improving health literacy among adolescents and the public through school and social media platforms, and 3) providing full reimbursement of the gender-neutral HPV vaccine, as a strong signal that this vaccination is essential.

The discussions on HPV infections control focused on the need to: 1) encourage HCPs to facilitate patient data collection to support performance assessment of the national cervical cancer screening program, 2) advance the transition from cytology to HPV-based screening, 3) improve cancer prevention training and awareness for all HCPs involved in screening, including midwives, 4) identifying patient barriers to invitation acceptance, and 5) promoting urine or vaginal self-sampling screening techniques to improve acceptability, while establishing appropriate follow-up strategies for HPV-positive women. This report covers some critical findings, key challenges, and future steps to improve the status of HPV prevention and control measures in the country.

## Introduction

The HPV Prevention and Control Board is an independent, international, and multidisciplinary group of experts created in 2015 to provide evidence-based guidance and reflection on strategic, technical, and policy issues regarding the implementation and sustainability of human papillomaviruses (HPV) prevention and control programs. The Board aims to multiply and disseminate relevant information on HPV prevention and control to a broad array of stakeholders. It contributes to the control of HPV infection, prevention, and screening strategies of HPV-related cancers by organising two meetings every year; a “technical meeting” covering practical and technological topics such as vaccine characteristics, vaccine safety, screening technologies and landscape, treatment strategies, the role of healthcare providers in vaccination programs, and dealing with anti-vaccine messages [1–3]; and a “country meeting”, covering a strengths, weaknesses, opportunities, and threats (SWOT) analysis of s in a particular country or region, usually chosen due to challenges in the programs’ implementation [4]. The fourth country meeting was held in France, following successful meetings in Denmark (2016), Ireland (2017), and Colombia (2018). The decision to focus on France was influenced by the progress of the country in HPV prevention and the recommendations of our HPV Board advisors.

The eleventh meeting of the HPV Prevention and Control Board (HPV-PCB) was held in December 2021 as a hybrid meeting due to SARS-CoV-2. This report summarises the discussions and lessons learnt from a country meeting that discussed the situation of the HPV prevention and control programmes in France. The objectives of the meeting were:

- The healthcare system, prevention and control measures in France
  - ◦ Review how prevention and control programmes are organised within the French healthcare system
  - ◦ To provide a summary of the epidemiology, burden of disease, and surveillance related to HPV and HPV-related cancers in France
- Cervical cancer screening and Treatment in France
  - ◦ To give an overview of the current control measures in France
  - ◦ To discuss successes and challenges related to HPV screening and treatment
  - ◦ To review the possible implementation of alternative screening methods and strategies, such as self-sampling
- HPV vaccination in France
  - ◦ To give an overview of the evolving HPV vaccination program
  - ◦ To discuss successes and challenges related to HPV vaccination
  - ◦ To discuss the state of vaccine confidence, associated challenges, and possible solutions to increase vaccine coverage rate
  - ◦ To discuss the impact of Covid-19 pandemic on vaccine confidence and review the change in perception, beliefs, and opinions toward vaccination
  - ◦ To discuss the efforts and stakeholder participation in social mobilisation and creating awareness
- The various stakeholder perspectives
- The way forward

## The healthcare system in France

### Organisation of prevention and control programmes within the French Healthcare system

France, which has a population of around 67 million people, has a health expenditure of 4700 US dollars per person, equal to 12.3% of the French GDP. Cardiovascular diseases and cancer are the leading causes of death, whereas, before Covid, infectious diseases accounted for less than 2% of deaths. Regrettably, there is inequality in incidence and mortality rates, with some regions having higher premature mortality. Inequalities in the coverage of the HPV vaccination across departments are attributed to medical density and unemployment rates, among other factors.

The Ministry of Solidarity and Health runs various organisations that cover the funding and provision of health services. Among these organisations, the High Council for Public Health (HCSP) provides the authorities with prospective reflection and counselling on public health aspects, contributes to building a global and organised paediatric health policy, and contributes to elaborate and follow-up pluri-annually the national health strategy. In parallel, the National Health Authority (Haute Autorité de Santé, HAS) is an independent public authority that provides recommendations on professional practices and is involved in the organisation of care and public health. The HAS has several tasks, including 1) evaluation of health products, medical devices, and medical acts in order to allow their reimbursement, 2) recommendation of best practises and elaboration of public health recommendations, and 3) measurement and improvement of hospitals, clinics, outpatient care, and social and medico-social centres. The National Cancer Institute (Institut National de Cancer, INCa) is a state agency for health and scientific expertise in oncology, responsible for coordinating actions in the fight against cancer. INCa was created in 2004 and falls under the joint supervision of the Ministry of Solidarity and Health and the Ministry of Higher Education, Research, and Innovation. Its missions are to coordinate actions in the fight against cancer; to initiate and support scientific, medical, technological, and organisational innovation; to contribute to the structuring of the organisation of screening, care, and research; to provide expertise; to produce, analyse and evaluate data; and to promote the application of knowledge and good practices.

The National Public Health Plan 2018–2022 aimed to implement a health promotion policy, including prevention, in all settings and throughout life. This aligns well with the ten-year strategy (2021–2030) for the fight against cancers, which emphasizes improved prevention as the first axis in its plan to fight against cancers. One step towards prevention is the introduction of a national cervical cancer screening programme. To increase efficiency, the scale of the organisation has moved from departmental to regional. Despite this, many actors with different missions are involved, which can negatively impact efficiency.

The Ministry of Health has the political responsibility for primary prevention through vaccination. However, the organisation falls under the Chief Medical Officer (Direction Générale de la Santé, DGS). The French NITAG, the Vaccination Technical Committee (Comité technique des vaccinations, CTV), is a consultative body now under the aegis of HAS, acting after a referral from the DGS, works by assessing the strategic need for vaccination and proposing a recommendation supported by a medico-economic assessment. Based on these recommendations, the Ministry of Health will decide whether a vaccine should be integrated into the National Immunization Program.

### Organisation of the French Vaccination Technical Committee

In 2017, the CTV was moved from the HCSP to the HAS. The CTV proposes recommendations on vaccines. Next, the Transparency Committee performs an evaluation of the vaccine in terms of medical benefit at the population level and comparison to previous similar vaccines. Subsequently, the Economic and Product Health Committee decides on the amount to be reimbursed (35% or 65%). It discusses the public price, including negotiation with French public insurance (Social Security) representatives and the industry, on the acquisition price. The CTV also advises on immunisation schedules and mandatory vaccinations. CTV members are selected by obeying strict rules to prevent conflicts of interest.

As an example of the decision-making process for HPV recommendation and advice process, first, as soon as EMA granted the marketing authorisation, CTV analysed in 2007: 1) the incidence of cervical cancer, 2) the average age at first sexual intercourse, 3) the aetiology of HPV in precancerous lesions and cancer 15–25 years after infection, 4) the efficacy of the Gardasil vaccine against HPV infection and cervical lesions, 5) the presence or absence of side effects, 6) cost-effectiveness studies related to its administration, 7) the impact of vaccination on cervical cancer screening by cytology, and finally 8) emphasised the value of screening. The voting members of the CTV proposed a recommendation for girls only with Gardasil (later followed by Cervarix) vaccination before the age of first sexual intercourse (14 years), with a catch-up up to the age of 23. Based on this, the Transparency Committee and the Medico-Economic and Public Health Committee decided on the public price and a reimbursement of 65% of the HPV vaccine by public insurance ("Sécurité Sociale”).

In 2019, based on the low coverage of the vaccine in girls and men who have sex with men (MSM), a recommendation was issued to vaccinate boys between 11 and 13 years of age with two doses, boys between 14 and 19 years of age and MSM up to the age of 27 years of age with three doses, without changing the level of reimbursement.

### Mandatory vaccination in France

Until 2017, three vaccines were mandatory in France: diphtheria (since 1938), tetanus (since 1940) and poliomyelitis (since 1964). Due to various media-divulged myths and rumours, such as hepatitis B vaccine association with multiple sclerosis, pertussis's vaccine association with sudden infant death, and the use of aluminium salt-based adjuvants with macrophage myofascitis, the French population has developed concerns over the years on vaccines safety, and association of vaccines and vaccine components with adverse events. According to a report on vaccine confidence, the vaccine hesitancy rate in France is 45.2%. The overall average rate of vaccine hesitancy in all the countries surveyed (67 countries) was 13% [5]. A population-based consultation led to the decision to increase the number of mandatory vaccines to 11, all of them already routinely recommended for children under the age of two, strongly supported by the consensus of medical societies. Eventually, the law was passed on 25 January 2018, stating that all infants born after 1 January 2018 must be vaccinated to have access to a daycare centre or official private nurse care, with sanctions for parents who refuse. In light of parental noncompliance with legal obligations that jeopardise the well-being of their child, parents or caretakers may face legal repercussions, including a potential prison term of up to 2 years and a financial penalty of up to 30,000 euros. In addition, Doctors providing false vaccination certificates and parents using those may face up to 3 years of imprisonment and a fine of 45,000 euros. These measures also relied on vaccine availability and the number of visits per child, already authorised and reimbursed, up to 2017.

A rapid and significant increase in vaccine uptake was observed in 2018 when the mandatory vaccine policy came into effect. An increase in hepatitis B was observed. Furthermore, the uptake of the MMR and HPV vaccine increased in children born before the policy was applied, suggesting an associated contribution, among other causes. Moreover, mothers had a more favourable opinion about vaccinations and felt reasonably or well informed about them. Additionally, in 2019, the upward trend for vaccine uptake continued [6]. And not only parents but also GPs and paediatricians felt more confident after the extension of mandatory vaccination, as vaccination was easier to explain to parents and be accepted.

### Surveillance of cancer in France

The first French cancer registry was established in 1975. In 1997, the French Network of Cancer Registries (FRANCIM) was founded, with a centralised database created one year later. FRANCIM encompasses general and organ-specialised registries, including four overseas registries (Guadeloupe, Martinique, Guyana, and Reunion) and two pediatric registries. Since then, the network has grown to cover 26 departments (out of 96 metropolitan departments) and nearly 25% of the French population.

The key aspects of any cancer registry are completeness, comparability, and quality to ensure adequate data collection for surveillance and research. FRANCIM published the latest data on cancer incidence in France in 2019 [7]. These data show the incidence of HPV-related cancers in 2018, including 5000 cases of oropharyngeal cancer (1200 cases in women, 3800 cases in men), 2000 cases of anal cancer (1500 cases in women, 500 cases in men), 1000 cases of vulval or vaginal cancer and 450 cases of penile cancer. Yearly, about 35,000 conisations are performed, 3000 women are diagnosed with cervical cancer, and 1000 women die of cervical cancer.

The age-standardised incidence trend shows a gradual decline in both incidence and mortality of cervical cancer since 1990, when registries included cervical cancer burden as a health indicator, most likely due to cervical cancer screening practices. However, the incidence rate trends by age show a plateau for women in their fifties and sixties, with a slight increase in recent years. This may be due to changes in sexual behaviour, and suboptimal screening practises in these age groups. Similarly, the five-year survival rate is decreasing, which is mainly associated with lower survival rates in women over the age of 60, in whom cervical cancer is found at a later stage because of suboptimal screening practices. With these data, France scores in the middle for the age-standardised incidence rate of cervical cancer compared to other European countries [8]. Interestingly, analysis of trends in anal cancer incidence shows an increase in both women and men, especially in the older age groups.

### Discussion

At the end of the session, several points were raised for discussion.

Does the 65% reimbursement impact the use of HPV vaccines?

The whole French population is covered for health costs, with over 90% of the French population paying for complimentary private insurance, which will reimburse up to an additional 30% of the 65% already covered by public insurance, thus covering at least 95% of the cost of all vaccines, including HPV vaccine. The socio-economically deprived population benefits from couverture médicale universelle (CMU), covering 100% of all medical costs, including vaccines and hospitalisation. When patients within the recommended age group are brought to any vaccination centre by their parents, they will have free access to vaccination. Therefore, we believe that the economic impact is not an urgent factor to consider as a barrier or burden to a parent seeking HPV vaccination for their children.

What factors are associated with the described decrease in cervical cancer five-year survival rates?

The decline in survival in cervical cancer is likely due to the paradoxical effect of screening; women diagnosed with cancer during screening are more likely to have early-stage cancer, whereas those women who do not participate in screening are more likely to be diagnosed at an advanced stage. Looking at stage-specific survival should solve this problem.

Did making vaccines mandatory lead to increased vaccine confidence? Medical societies were collectively very positive, and since the public trusted them well, they also represented a positive buffer between public health authorities, politics and the public. General practitioners and paediatricians were also in favour because the mandatory process helped parents understand the benefits of vaccination. Finally, public health authorities backed the process and developed good websites with adequate information on vaccination. In Italy, where in 2017, ten vaccines were made mandatory, vaccine confidence also increased soon after, which was evident once the Covid vaccines became available. This template may be helpful for Japan, where there is broad medical support for vaccination, but no public support. In Turkey, several vaccines are mandatory, and vaccine coverage of such vaccines is greater than 95%. GPs who do not reach the targets are not reimbursed (< 70%), whereas higher vaccine coverage provides a higher reimbursement.

One major concern about recommending new vaccines is that they must go through all legal aspects to become mandatory, which is a lengthy process. Moreover, as long as it is optional, it may be perceived as less important, impacting vaccination coverage.

## HPV Epidemiology and cervical cancer screening in France

### Surveillance of HPV in France

For optimal surveillance of HPV, both to support the introduction of the HPV vaccine and cervical cancer screening, a national reference centre was set up in 2009. Since 2017, it has been hosted by the University Hospital of Besançon. France's network includes other university hospitals, general hospitals, and private pathology laboratories. The French reference centre is also part of the international network of HPV reference laboratories, coordinated by Professor Joakim Dillner (Karolinska, Sweden).

The reference centre has four missions: to provide expertise in the validation of kits, collection media, and sampling devices; to provide counselling, education, and information on public health policies to the Ministry of Health and other parties; to perform epidemiological surveillance at the national and international level; and to be alert to signal problems at an early stage.

An example of epidemiological surveillance is the distribution of HPV in oral and anal self-samples from MSM [Pretet, manuscript submitted]. In anal samples, the prevalence of HPV was 82%, with multiple infections in 58%. In oral samples, the prevalence was 11%, with multiple infections in only 1% of the samples.

Technically, the preservation of cervical specimens on FTA cards showed that HPV DNA could be safely recovered after 54 months of storage at room temperature. A concordance of 97% was achieved between the HPV genotypes detected in the initial cervical samples and on the FTA cards 4.5 years later [9].

Another study estimated the prevalence of the different HPV genotypes in 600 high-grade cervical lesions in patients born between 1972 and 1993. The goal is to determine whether the prevalence of HPV16 and HPV18 is lower in the population born between 1983 and 1993 and potentially exposed to HPV vaccination compared to those born between 1972 and 1982 who were not exposed to vaccination [NCT04167501]. Other studies will evaluate vaccine effectiveness in female students and the HPV genotype distribution in high-grade cervical lesions and cancer in French Guyana.

### Practical organisation of the French cervical cancer screening system.

France has had opportunistic screening since the 1980s. A few pilot programmes were organised and launched in the 1990s, but a nationwide organised screening was not launched until 2014 as part of the Third National Cancer Plan. Currently, national screening coverage is around 60% for women between 25 and 65 who have ever been screened, although lower in overseas territories. Even within France, there are important social inequalities in access to screening.

The objective of the screening programme is to reduce cervical cancer incidence by 30% through improved coverage, consistent follow-up of screen-positive women, and reduced inequalities in access to screening. The program is coordinated at a regional level by seventeen regional cancer screening coordination centres (CRCDCs) responsible for the invitations, appropriate follow-up, quality assurance and communication. The general practitioner (GP), gynaecologist, or midwife performs spontaneous screenings when women come to the gynaecologist consult. The programme actively invites women who may not naturally seek consultations with their general practitioner/gynaecologist to participate. The invitation stresses that participation is free of charge and allows for opting out. In the event of a positive result without follow-up exams, the healthcare provider (HCP) or the woman herself is contacted. As of June 2021, three years after the program was launched, only 13 of the 17 CRCDCs responded to the INCa activity questionnaire. From this, only seven CRCDCs have invited all eligible women, nine report difficulties in reaching HCPs, and test results are consistently collected in only three CRCDCs. The lack of a national information system hampers implementation, further aggravated by GDPR constraints. Subsequently, INCa started working on a project to collect all test and histology results at a national level. A national committee was formed to create and launch guidelines for self-sampling in women who have yet to respond to previous invitations.

### Cervical cancer screening. The transition from cytology to HPV-based screening

France is transitioning from cytology-based to HPV-based screening, as recommended by HAS in 2019. HPV testing has a higher sensitivity and negative predictive value and will facilitate an extended screening interval of five years after an HPV-negative result. After an HPV positive result, reflex cytology will be performed, and when lesions are found, the women will be referred for colposcopy. If cytology does not show lesions, HPV testing is repeated after one year.

To move toward a transition, first, a validated screening assay must be selected for the introduction of HPV-based testing. Initially, five tests were selected, which have been approved by the US-FDA [10], a protocol for clinical validation was established [11], and several tests were found suitable for screening [12]. Secondly, an appropriate test for triaging HPV-positive women is required as HPV-based testing has low specificity. The optimal triage test should find all high-grade lesions while avoiding unnecessary colposcopy and overtreatment [13]. Other tests are under evaluation, including cytology staining for p16/Ki67 and methylation assays. With increased use and lower cost, whole-genome sequencing may simultaneously provide information on HPV genotype and methylation targets.

Finally, integrated screening is necessary, including management/follow-up of HPV-positive women, with an organised administration to facilitate call and recall, as most cervical cancer cases occur in women who do not participate in screening, and strategies to increase screening coverage, e.g., by self-sampling as an alternative for pelvic examination. Eventually, screening and vaccination programmes will have to be integrated, as the entry of vaccinated women into the screening age cohort will negatively impact the positive predictive value of cytology.

Unfortunately, co-testing is often used in France without a clear benefit; it is more expensive and does not provide additional reassurance relative to an HPV-negative test, with only five cases per million per year based on the cytology addition.

### Challenges in the cervical cancer screening program in France

The French screening algorithm is in line with the European guidelines for quality assurance in cervical cancer screening [14], with cytology-based screening for women up to the age of 30 and HPV-based screening for women over the age of 30. To find the best screening method for cervical cancer, a comparison was made between conventional cytology (Pap smear), visual inspection with acetic acid (VIA), HPV nucleic acid (HPV DNA) testing, and combinations of these tests. The HPV nucleic acid test was superior in all cases, whether used alone or combined with other methods [15]. However, the real-life deployment of evidence-based organised screening has been complex. In France, the gynaecologist does most screening in private practice, with inappropriately short intervals between tests. Although this opportunistic way of screening reaches around 60% of the population, a more systematic, organised approach with central coordination would benefit more people. Furthermore, the option to do co-testing is chosen too frequently, facilitated by the design of the request form. It is also proposed by pathology labs.

Another challenge is the obligation to confirm that the woman has agreed to share her data. The gynaecologists miss the opportunity to confirm this on the questionnaire; therefore, the data cannot be shared and is subsequently lost for the screening program's quality assurance (QA).

To overcome these challenges, it is necessary to convince HCPs to apply these recommendations. An option would be not to reimburse screening tests after 30 years of age if more than 20% of the users have had two tests (i.e. co-testing). Additionally, it is important that healthcare professionals prioritise the confirmation of patient consent in transmitting anonymous data for program quality assurance. Lastly, it is important to encourage women to participate in screening when invited.

### Self-sampling as an alternative/additional strategy in France.

While a screening program should theoretically reduce the incidence of cervical cancer by 90%, because of the low coverage in France, this target has not been reached. The third French Cancer Plan aimed to reduce incidence and mortality by increasing coverage and making it more accessible to vulnerable populations through self-sampling. A French study showed that offering vaginal self-sampling is a cost-effective way to increase participation, which was higher in the 'self-sampling' than in the 'no intervention' group and the 'recall' group [16]. Furthermore, most women with positive HPV test results underwent the recommended triage with conventional Pap smear. As an even less invasive technique, urine testing is only used in research settings. In the CapU study to evaluate the acceptance of a urinary HPV test, letters were sent to 5000 women aged 40–65 years who had not had a Pap smear in the previous three years [17]. Of the 771 samples received, 687 were analysed, and high-risk HPV was detected in 29 women, of whom 28 had a Pap smear or colposcopy done.

The cytological results showed nine abnormal Pap smears, including three cases of cervical intraepithelial neoplasia grade III (CINIII) lesions upon histology. A follow-up study proposed urinary HPV testing to 13,535 women aged 35 to 65 years without a recent Pap smear. The participation rate was 15.4%. Of the 1,915 samples analysed, 190 were HPV positive. A satisfactory gynaecological follow-up was observed for 92% of the HPV-positive women [18]. Twenty-three abnormal smears were observed, and eight high-grade lesions were diagnosed after colposcopy and biopsy. This indicates that home HPV urinary testing may be an alternative for women reluctant to have a Pap smear and thus extend screening coverage. To evaluate the efficacy of urine or vaginal self-sampling invitation strategies in cervical cancer screening, a randomised controlled trial has been set up with three arms of 5000 women each: The first group received a traditional invitation letter, while the second group received an invitation letter along with a vaginal self-sampling kit. The third group received an invitation letter and a urine self-sampling kit [19]. The 2021–2025 Cancer Plan aims to achieve 70% screening coverage, including self-testing devices.

### Head and neck cancers in France

Globally, 900,000 new cases of head and neck cancer (HNC) occur each year. The main risk factors for HNC are tobacco and alcohol, betel quid, and viruses, including HPV and EBV, indicating that most cases are preventable. France is among the countries with the highest incidence of HNC in Europe, and the same is true for the subset of oropharyngeal cancer (OPC). Two recent studies, one based on HPV mRNA testing in HPV DNA positives [20] and one based on HPV DNA testing in p16 positives [21], showed that 27 to 38% of all OPC are HPV positive.

By analysing trends from 1980 to 2012 using an age-period cohort model based on data from 11 French cancer registries, the age-standardised incidence rate of OPC among men decreased slightly. Among women, the age-standardised incidence rate increased [22]. The pattern is consistent with observations made in other countries, with studies of HPV prevalence in OPC and the evolution of sexual behaviour in France. Furthermore, an assessment of 1230 OPC samples from the Gustave Roussy Cancer Center shows an increase in the HPV-attributable fraction from around 30% to around 50% of cases. A large global epidemiologic study has been set up to estimate the current and recent past HPV burden in oropharyngeal and non-oropharyngeal HNCs, to provide robust estimates of HPV attributability by anatomic site in participating countries [23].

The general lack of awareness of the association between HPV and HNC is a major concern, as it can lead to misdiagnosis and, consequently, delay diagnosis. Considerable efforts are needed to increase awareness among the medical community and the general population.

### Discussion

At the end of the session, several points were raised for discussion.

Cytology is often used for younger women. Due to increased vaccination coverage, could HPV-based tests not be performed in this age group, as fewer HPV positives are expected in vaccinated women?

Are HCPs checking the co-testing box because they are insufficiently informed about reflex cytology testing?

They are generally aware of reflex testing but have a financial incentive to request co-testing. The possibility should be considered to remove the box for co-testing completely. A reimbursement policy worked very well in Slovenia to stop HCPs from requesting the wrong tests. This may not work well in France and may be perceived as provocative, as the patient may side with the gynaecologist due to the long-standing relationship.

To choose the preferred way to be screened, women must be well informed of the benefits and conditions of self-sampling.

With so many private labs, how is quality assurance performed?

There is no obligation to have QC/QA, except for clinical pathology, for which accreditation is necessary.

Are there differences between HPV-positive and HPV-negative HNC patients? There is a big difference in overall survival, with HPV-positive cancer patients having a much better chance of survival and smaller tumours in the first place, which makes it possible to have robotic surgery. Regrettably, no screening for OPC is possible, making it even more relevant to raise awareness and ensure that cancers are diagnosed at the earliest possible stage. In the longer term, E6/E7 serology might be an option for screening, but this has yet to be confirmed.

For anal cancer, which is also HPV related, the incidence of the disease is increasing in France, both in men and women, especially in older women. Screening could focus on high-risk groups such as MSM and people living with HIV. However, anal cytology does not perform well, and high-resolution anoscopy is quite complex and cannot be easily performed. A future session may be needed to discuss this topic in more detail.

## HPV vaccination in France

### Development and implementation of the HBV and HPV vaccines

Many similarities exist between the development of the hepatitis B virus vaccine and the HPV vaccine: the long phase between antigen discovery and industrial production of the vaccine, vaccine hesitancy due to misinformation or debatable decisions by health authorities, and a target age group (the second decade of life, 10–20 years of age) which is more prone to autoimmune disorders. For Hep B vaccination in France, this trend was only partially reversed by introducing universal immunisation of infants in 2003, followed by free-of-charge vaccination in 2009. Finally, making the vaccine mandatory in 2017 was the most efficient incentive. The vaccination coverage rate is now around 90%.

For the HPV vaccine, the self-assembly of the L1 structural protein into virus-like particles with the same morphology and conformational epitopes as the virus itself and the production of L1 in insect cells or yeast has paved the way for large-scale antigen production. Four vaccines are now commercially available, and effectiveness of these vaccines against cervical cancer has been shown [24–26]. Although universal and reimbursed immunisation for girls between 11 and 14 years of age was introduced in 2013, the vaccination coverage rate has remained low, below 30% in 2020. While making several infant vaccines mandatory had a positive impact also on HPV vaccination, there is ample room for further improvement. Although it took 30 years, HBV vaccine coverage in France is now satisfactory. Applying similar solutions to HPV immunisation may help reach a vaccine coverage rate similar to what has been observed in other high-income countries for many years.

### HPV vaccination in France

The initial vaccine recommendation, in 2007, was aimed at 14-year-old girls, with a catch-up vaccination of girls between the ages of 15 and 23 years who had not started sexual activity or were within the first year after initiation of sexual activity. This reference to sexual activity may have confused parents and led to hesitancy. In March 2007, the first recommendation for the HPV vaccine only mentioned the 4vHPV vaccine [27], which may have caused uncertainty about the preference for the 2vHPV vaccine and HPV vaccination in general. HAS issued an updated recommendation in 2010 [28], which included the use of the 2vHPV vaccine. The initial exclusion of the 2vHPV vaccine was due to a lack of effectiveness data at the time. Unfortunately, this led to more doubt and reluctance to get vaccinated.

Nevertheless, in 2010, a vaccination coverage rate of 60% was achieved in the 17–20-year age group, receiving at least one dose. As a result of constantly updated messages from health authorities, a media crisis ensued in 2011. Several social media channels claimed that vaccinating with the 4vHPV vaccine (Gardasil) has caused adverse events following vaccination, leading to a 50% drop in the number of vaccinated girls. During that year, French national bodies conducted a study involving 1.8 million French girls. The study provided reassuring information on vaccine safety. The number of autoimmune diseases in vaccinated girls was not higher than in unvaccinated girls, except for some increase in Guillain–Barre syndrome. The absence of association between the incidence of any autoimmune disorders was confirmed in a study with a slightly different design [29]. This led to the conclusion, in 2013, that data from the international and French literature did not show an increase in the incidence of autoimmune diseases or, more specifically, of multiple sclerosis after vaccination with Gardasil.

HPV vaccination coverage is steadily increasing; however, it is far from WHO's 90% vaccination target to eliminate cervical cancer as a public health concern by 2030. Additionally, geographic disparity has been brought to attention in regions with low coverage scattered across the map, including overseas departments.

### Role of HCPs in HPV vaccine hesitancy in France

The French population trusts doctors more than any other profession. Hence, physicians play a pivotal role in the population’s vaccination, as their support of the official recommendations strongly influences the acceptance or refusal of the vaccine.

Qualitative interviews with French physicians (GPs, gynaecologists, and paediatricians) showed that two-thirds (19/28) of the participants were favourable to HPV vaccination; some opposed it (4), while others were reluctant to recommend it [30]. Three different ways to interact with patients on this topic were identified: informing and convincing, adapting to patients' opinions, and refusing compromise about vaccination. Five types of physicians using these strategies were identified: 1) dissidents (mistrustful of the healthcare system and HPV vaccine), 2) hesitant (finding it difficult to make up their minds about this vaccination), 3) laissez-faire (letting patients decide by themselves, but very favourable to HPV vaccination), 4) educator (very favourable), and 5) uncompromising vaccinators (refusing debate). Trust in stakeholders involved in designing and implementing HPV vaccination strategies influences physicians' judgment on HPV vaccination [30].

Initial and continuous training of medical students is essential to enable them to fulfil their role as vaccinators. A nationwide cross-sectional online survey was conducted among students from 27 medical schools in France regarding their vaccination education [31]. The survey covered their knowledge, attitudes, practises, and perceptions and assessed their perceived preparedness for their future practise as interns. Approximately a third of medical students felt unprepared for vaccination questions, especially in communicating with patients about side effects and strategies to respond to vaccine hesitancy. Practical training was associated with better-perceived preparedness [31]. Methods based on practical learning (case-based learning, clinical placements, and other hands-on techniques) will likely produce the best results and should be favoured to improve students' preparedness.

To conclude, the perspective of other healthcare professionals (pharmacists, nurses, midwives) in the vaccination process in France has to be considered, as is already the case for several other vaccines for individuals 18 years of age and older. The HAS recommended in June 2022 that this be expanded to people under 18 years of age.

### History of anti-vaccination sentiment in France

In all countries, resistance to vaccination is as old as vaccination itself. After the inoculation against smallpox, Edward Jenner introduced the first proper vaccine, immunisation with a viral strain of bovine origin. Soon after, rumours claimed that vaccinated humans risked developing cow-like body parts. This shows that social media is not needed to develop fake news and vaccine hesitancy, though they are powerful facilitators. Louis Pasteur, who developed the rabies vaccine, also faced fierce opposition from scientific colleagues (Michel Peter, spokesperson of the Academy of Medicine) and the media (Henri Rochefort, editor of the newspaper l’Intransigeant).

The Universal League of Anti-Vaccinators started in England in the late nineteenth century, but still exists today and is active in France: the Ligue Nationale pour la Liberté des Vaccinations.

Anthroposophy, founded by Rudolf Steiner at the beginning of the twentieth century, still has many followers in Europe and is opposed to vaccinations. Many Steiner-Waldorf schools still exist, mainly in Europe, where more than 70% of all these schools can be found.

Another force against vaccination is libertarianism, a form of ultraliberalism, especially regarding economy, anarchism, individualism, and isolationism. This movement denies the authority and power of the state and calls for the dissolution of the coercive social institution. For libertarians, mandatory vaccination is a violation of the body.

One of the main tools used to spread vaccine misinformation is false publication, with the 1998 Lancet publication on the association of autism and the measles vaccine as a prime example. After this publication, vaccination coverage decreased significantly, especially in the UK, causing measles outbreaks worldwide and returning vaccination coverage to levels before this publication took several years.

Another infamous example is the paper that suggested that the HPV vaccine causes cervical cancer. The author of this paper claimed to work at the Karolinska Institute, but no one with this name worked at this institute. In fact, this institute was among the first to show that the vaccine prevents cervical cancer [24].

In 2019, the WHO declared vaccine hesitancy one of the top ten threats to global health. Distrust and rejection of authority have been described as the main reasons for the reluctance towards vaccines [32]. Therefore, strategies must be developed to combat this phenomenon.

### Insights into HPV vaccine confidence in France

Although HPV infection is one of the most common sexually transmitted infections in Europe, HPV vaccination remains highly controversial in many countries, including France. Participants in European studies most commonly reported issues with the quantity and quality of information available about HPV vaccination, followed by concerns about potential side effects of the vaccine and mistrust of health authorities, healthcare workers, and new vaccines. Studies have found that low HPV vaccine uptake in France can partly be explained by concerns about the risks associated with the vaccines, their safety, effectiveness, and importance [33].

To explore HPV vaccination decision-making among mothers and adolescent girls in France, a study was set up in Paris, recruiting vaccinated and unvaccinated girls and their mothers for interviews and focus groups [34–36].

The main themes found to be associated with the decision-making process of adolescents and mothers for HPV vaccination were maturity, the influence of risk–benefit perceptions and trust of vaccines and stakeholders involved in the exercise of vaccination: 1) Maturity was shown to have an important role in HPV decision-making: Most adolescents felt not included in HPV decision making, as most of the time communication strategies are aimed at the caregivers or parents. In contrast, they can have the maturity to make informed decisions. This shows the need for individualised communication approaches with adolescents themselves to strengthen discussions and information on HPV vaccination [36]. 2) Risks and benefit perceptions differ between mothers and adolescents, with mothers' risks in line with media crisis controversies, such as adverse events following vaccination, and adolescents' more concerned about short-term side effects, such as fever and pain at the site of infection. The risk of caregivers transmitting their concerns and hesitancy to adolescents should be mitigated by ensuring that adolescents are informed through other sources, such as schools. Furthermore, communication to address mothers' concerns is also required [34]. 3) Trust in stakeholders involved in the exercise of vaccination in France is high; therefore, HPV vaccine confidence can be increased if doctors more commonly recommend vaccination. However, trust in health authorities is much lower, given broader trust issues associated with a lack of solid support in regards to HPV vaccination [35]. However, in France, legally, no vaccination can be administered to adolescents below the age of 18 without the consent of the parents. This may lead to the adolescent wanting to be vaccinated, but the parents disagree.

### HPV vaccination in schools; pilot project in Guyana

A project was set up in French Guyana to train local vaccinators and organise a school-based HPV immunisation campaign. French Guyana is in South America, with 90% of its area covered with forests, leaving a narrow coastline with all the infrastructure. Nevertheless, 20% of the population lives along the border of two rivers and can only be reached by pirogue. Cervical cancer is the second most common cancer among women and HPV prevalence rates are high, with limited access to cervical screening. Hence, immunisation is the prioritised line of prevention. As a first step, an information campaign was started to inform parents and pupils about cervical cancer prevention through immunisation and parental consent. Alongside this, professional training on HPV vaccination, including safety, effectiveness, and dealing with vaccine hesitancy. This training was provided to doctors, nurses, and midwives and was accredited by the National Agency for continuing professional development. On the first day of the campaign, 86 of the 246 registered girls were vaccinated. However, resistance emerged from local and church leaders, and in combination with the Covid pandemic, the campaign had to be stopped. The continuation of the project is planned for the first quarter of 2022, still with the objective of performing a vaccination program in all middle schools.

### Discussion

At the end of the session, several points were raised for discussion.

What is the role of fathers in the decision process?

Mothers are more important in decision-making, but fathers are also involved. Although it is generally felt that fathers do not actively participate in decision-making, they seem increasingly keen to do so.

How should trust be defined?

It is essential to highlight that results from proximity, therefore, at different levels, such as trust in the product, the HCP (associated with closer proximity), and the authorities (associated with distance proximity).

## Treatment of cervical and other HPV-related cancers

### Treatment of cervical cancer in France

Recent data indicate a break in the slope of a decrease in cervical cancer incidence and mortality, possibly caused by more aggressive tumours, which are not detected by screening, occurring in young unvaccinated women.

Treatment of cervical cancer is discussed in multidisciplinary teams and depends on tumour size (as observed by MRI) and lymphovascular space invasion status (LVSI, as observed by Pet Scan and/or lymphadenectomy). For women eligible for conisation (stage IA1), those who are LVSI negative, treatment is based on the histologic margins. Those with negative or wide histologic margins can be followed up with HPV detection, while women with positive or small margins undergo radical hysterectomy type A. Women who are LVSI positive undergo pelvic lymphadenectomy of the sentinel nodes. If the nodes are negative, the woman is treated with radical hysterectomy type B. If the lymph nodes are positive, the case is considered advanced stage, with treatment schedules selected accordingly. This is also the case for stages IA2 and IB1. Radical hysterectomy is no longer an option for larger tumours (2–4 cm, stage IB2), and women with negative lymph nodes are treated with brachytherapy (± external radiotherapy). For stages IB3 and IVA, concomitant radiochemotherapy is proposed, except stage IVA with the risk of fistula, in which pelvic exenteration is performed.

In case of recurrence, detected with magnetic resonance imaging (MRI) and positron emission tomography (PET) scan, radiotherapy can be applied (if not applied during initial treatment). Otherwise, surgery (pelvectomy) is necessary. Chemotherapy for recurrences, e.g., using Bevacizumab or anti-PD-1, is currently under investigation.

Radical hysterectomy is performed according to the Querleu-Morrow classification [37], in which type A is only performed in women under 50 years of age, with a tumour less than 2 cm, stromal invasion less than 10 mm, and negative LVSI and lymph nodes.

In the case of pregnancy at the time of diagnosis, the length of pregnancy is important for the approach; after 24 weeks, treatment is postponed until fetal maturity for those stages that allow waiting (IA1/IB1).

### Uterine preservation in cervical cancer

A proportion of women with cervical cancer are confronted at an age where they still may want to have children, in which case they must be treated conservatively, however, without increased risk of recurrence compared to traditional treatment. Furthermore, the treatment must increase the chance of having healthy babies afterwards. The experience at the hospital of Lyon over the period 1986 – 2011 included 160 cases of radical trachelectomy in women with a mean age of 31.5 years [38]. The initial stages were IA1/IA2 in 24% and IB1 in 76% of the cases, with squamous cell carcinoma in 77% and adenocarcinoma in 22% of the cases. Tumour size was limited to below 2 cm in 81% of the cases.

Regarding safety, nine recurrences occurred, leading to six deaths, while two patients were disease-free after further treatment. The risk of recurrence was closely associated with lesion size, with a ten times greater risk if the diameter of the tumour exceeded 20 mm [39]. Pregnancies occurred in 80% of the women, and live births in 65% of them. The European Society of Gynecological Oncology (ESGO), the European Society for Radiotherapy and Oncology (ESTRO) and the European Society of Pathology (ESP) have jointly developed guidelines for fertility-sparing treatment, following the best available evidence and expert agreement [40].

It is still debated whether radical trachelectomy is safe in the case of LVSI; however, LVSI is not a prognostic factor for recurrence. Ultrastaging the sentinel nodes may help to find micrometastases that are contraindications to fertility preservation. Finally, to offer fertility preservation for more advanced cervical carcinomas, a study used neoadjuvant chemotherapy before radical trachelectomy in 19 women with a tumoral diameter of 29 to 51 mm (mean = 37 mm). After a median follow-up of 79 months, two early recurrences were observed and four healthy babies were born [41]. Studies with a more significant number of patients and adequate follow-up are required to validate this conservative approach and clearly define the indications of this treatment.

### Discussion

At the end of the session, several points were raised for discussion.

How can the observation that the decrease in incidence and mortality of cervical cancer in France have slowed down be explained?

This is mainly due to very aggressive tumours in younger women, with such a short period between infection and full-blown cancer that the screening programme cannot find them. These cancers are treated in the same way as other less aggressive cancers. Perhaps treatment needs to be tailored more accurately. The use of neoadjuvant chemotherapy can be important here. It may also be helpful in low- and middle-income countries, where surgery and radiation therapy resources are not always available.

Will new chemotherapeutic agents (e.g., Bevacizumab or anti-PD-1) be a game changer in the field of cervical cancer treatment?

Currently, these agents are only used in trials, and we are awaiting long-term follow-up results, but the early results look promising. In Switzerland, where there is more experience with anti-PD-1, the drug is active initially, but after 6–8 months, progression is observed again, making the drug applicable to a limited number of cases.

For women living with HIV, should high-grade lesions be treated more aggressively?

HIV is not an indication to treat more aggressively. Control of the HIV infection is more important than how lesions are treated. However, vaccination and screening for women living with HIV remain extremely important, as that may prevent the development of cancer in these women and in non-HIV-positive women.

## Important stakeholders in HPV prevention and control

### (Nearly) 20 years of Infovac-France: daily Q&A

Political decisions on health issues are generally slow, and, usually, the decision process is not linear, making it often difficult for HCPs to understand the reasoning. Often, yearly changes in the vaccination schedule contribute to misunderstanding and a difficult follow-up of the recommendations. Recommendations for vaccination schedules are often aimed at both the general population and specific groups. However, healthcare professionals must handle each case individually and address practical questions on the spot. As a result, it is crucial for these professionals to stay up-to-date with the latest information continuously. Unfortunately, health authorities have been inactive in providing continuous support to HCPs after the initial recommendations were made. Additionally, vaccine companies are unable and unwilling to fill this gap, as it could lead to perceived conflicts of interest and further mistrust.

Infovac-France was set up in 2003 to fill this void, as a platform of expertise in vaccinology, following the Swiss example. Initially, the focus of Infovac was on paediatricians, but this was expanded to GPs, pharmacists, nurses, and midwives. Though Infovac has an internet site open to the public, the platform of expertise itself is for HCPs only. The platform is independent and unpolitical, providing one-on-one advice but not recommendations on specific queries or questions regarding any aspect of vaccines and vaccination with shares valid documents and references if necessary. The platform receives around 8000 questions per year, and on average, 10 min are spent per question to provide evidence-based answers within 24–48 h. Infovac is open throughout the year, including weekends and bank holidays. Infovac acts as a finger on the pulse regarding any aspects of vaccines and vaccination because of the questions that are received, including hesitancy in HCPs and, through them, the general public. During annual meetings, the Infovac experts gather to receive activity feedback and discuss uncertainties, grey areas, or topics requiring consensus. The number of questions has steadily increased from 30–50 per week in the early years to 100–150 per week in 2021, making it a heavy burden for the experts who generally do this next to their regular job. However, experts are compensated for the work they have performed. As many experts have been active since the start of Infovac, new members are needed, and the total number of members may have to be increased to reduce the workload.

The most frequently visited topics on the Infovac website are the frequently asked questions list and the pages dedicated to vaccine availability (shortage) and vaccine hesitancy.

### Optimising HPV vaccination communication to adolescents

Several issues still need to be resolved to optimise information and communication on the HPV vaccine with adolescents: whether to preferentially speak about cancer or genital warts; how to address HPV vaccine safety, given the suggested link with GBS in France (which is not reproduced in other countries); whether and how to address herd protection; how to present information on vaccine coverage; how to address currently insufficient vaccine coverage in France; and whether to present HPV as a sexually transmittable infection.

Discrete choice experiments can be performed to quantify the weight of these determinants in vaccine acceptance. Participants can be asked to decide for or against vaccination in hypothetical scenarios containing various levels of attributes described, as previously shown in university students [42] and healthcare workers [43]. Girls and boys (aged 13–15 years) participated in an internet-based study to identify optimal statements regarding a vaccination program, including vaccine characteristics [44]. In ten hypothetical scenarios, participants decided to sign up or not to participate in a school-based vaccination campaign against an unnamed disease. Scenarios included different levels of four attributes: the type of vaccine-preventable disease, communication on vaccine safety, the potential for indirect protection, and information on vaccine uptake among peers. One scenario was repeated with an additional mention of sexual transmission.

When the conversation focused on the cancer prevention benefits of the HPV vaccine, the participants were more willing to accept it. However, when the conversation focused on protection against genital warts, it did not motivate the participants. Girls were more likely to accept the vaccine for indirect protection, but this was not the case for boys. It was also observed that positive reporting phrasing, such as reporting that "more than 80% of young people in other countries got vaccinated", motivated vaccine acceptance compared to using "insufficient coverage,". Finally, the notion of sexual transmission did not influence acceptance [44]. This study shows that the communication of the HPV vaccine to adolescents can be tailored to optimise the impact of promotion efforts. It's important for the information to be relevant to cancer, even if it pertains to a time far ahead in the future. The safety of the HPV vaccine is best addressed as follows: No suspicion of a severe side effect has been scientifically confirmed. Furthermore, it is helpful to mention collective protection, the potential for elimination, and the high coverage of the HPV vaccine achieved among adolescents in other countries. Note that the message tailored to adolescents may not be the best message for parents, which is currently under investigation.

## Lessons Learnt & the way forward

### HPV Vaccination

Implementing the HBV and HPV vaccines in France faced similar challenges, such as hesitancy driven by misinformation or controversial decisions and targeting young adults aged 10–20. Although universal infant immunisation followed by free vaccination improved the low coverage of the HBV vaccine, mandatory vaccination in 2017 was the most effective measure, leading to 90% coverage. Despite the introduction of a free HPV vaccine for girls aged 11–14 in 2013, the coverage rate remained low at under 30% in 2020. Leveraging strategies seen as successful in other vaccine programs may help France achieve higher coverage rates for HPV vaccinations.

The initial recommendation for HPV vaccination in France in 2007 targeted 14-year-old girls, with catch-up vaccination for girls aged 15–23 who had not started sexual activity or were within the first year after initiation of sexual activity. The reference to sexual activity may have caused confusion among parents and led to hesitancy. Additionally, the first recommendation focusing only on 4vHPV vaccine was perceived as preference, over the 2vHPV vaccine. This may have cast doubt on the general preference for HPV vaccination, and the update of this recommendation in 2010 only added to the doubt and reluctance to vaccinate. However, the vaccination coverage rate increased to 60% in the 17–20 age group that received at least one dose in 2010. In 2011, a media crisis occurred due to unclear messages from health authorities, with several social media channels claiming that the 4vHPV vaccine (Gardasil) had caused adverse events following vaccination, resulting in a 50% drop in the number of vaccinated girls. However, studies conducted by national bodies, including 1.8 million French girls, showed that the number of autoimmune diseases in vaccinated girls was not higher than in unvaccinated girls, leading to the conclusion that data from the international and French literature did not show an increase in the incidence of autoimmune diseases or multiple sclerosis after vaccination with Gardasil. Despite steadily increasing HPV vaccination coverage, it is far from the WHO 90% vaccination target to eliminate cervical cancer as a public health concern by 2030, and geographical disparities have been identified in regions with low coverage.

Physicians play a crucial role in the vaccination of the French population, as their support of official recommendations strongly influences vaccine acceptance or refusal. A study of French physicians (GPs, gynecologists, and pediatricians) showed that two-thirds (19/28) of the participants were favourable to HPV vaccination, some opposed it (4), while others were hesitant about recommending it. The physicians' judgment was influenced by their trust in the stakeholders involved in designing and implementing the HPV vaccination strategy which further reinforces the importance of HPV awareness at this level. To enable physicians to fulfil their role as vaccinators, strong training of stakeholders involved in vaccination (medical students, GPs, midwices, gyneacologist, peadiatricians and preservice healthcare professionals) is essential. Practical training was associated with better perceived preparedness, and methods based on practical learning are likely to produce the best results and should be favoured to improve student preparedness. Lastly, in February 2023 French authorities announced reimbursed recommendation of HPV vaccination for adolescents (boys and girls) between the ages of 11 and 14 and up to 19 years of age for catch-up vaccination. Thus, further strengthening the overall importance of HPV vaccination in preventing HPV-related cancers.

### Cervical cancer screening

Cervical cancer screening is undergoing a paradigm shift from a cytology-based approach to an HPV-based approach in women aged 30–65 years. The successful implementation of this transition requires the participation of all stakeholders involved in the screening process, including healthcare authorities, medical societies, and healthcare professionals. Co-testing does not improve HPV-based testing alone, and thus it is essential to apply the screening algorithm recommended by the relevant regulatory bodies (i.e., HAS). Furthermore, the use of clinically validated HPV tests and continuous quality assurance laboratories accreditation with international standards ISO15189 is necessary to ensure the best performance of the screening process.

Regional Cancer Screening Coordination Centers (CRCDC) have a crucial role to play in organising and coordinating the screening process. The next step in cervical cancer screening is to consider how to screen vaccinated women. It remains unclear whether women below 30 years of age should still be screened by cytology, given the decreasing prevalence of HPV-positive cases. Innovative molecular tests, including viral load, genotyping, and methylation, may prove useful in triaging HPV-positive women more accurately than cytology. However, more large-scale studies are needed to evaluate the efficacy of these tests.

Guidelines for the use of self-sampling for non-respondent women are now available. Self-sampling has the potential to increase screening coverage, and it is imperative to involve regional CRCDC in its implementation and follow-up. Large-scale communication campaigns are necessary to encourage women's participation in the organised cervical cancer screening program, which, combined with vaccination, is key to eliminating cervical cancer.

This manuscript provides a comprehensive overview of France's HPV prevention and control programs up to December 2021, the date when the meeting took place. We acknowledge that the situation in France has changed since the meeting. However, the overview of the HPV prevention and control history in France up to December 2021 encompasses many lessons learned, which have the potential to contribute to and leverage opportunities for countries and regions that are optimising their prevention and control programs. Specifically, the events leading up to and following the implementation of 11 mandatory vaccinations are unique. This meeting report emphasises the importance of political determination, the power of stakeholder consensus (and the mobilisation process to obtain this consensus), and the subsequent outcomes as significant lessons learned that can serve as blueprints for other countries.

## Data Availability

All the presentations of the meeting report are published on the website (http://www.hpvboard.org) after speakers’ approval.

## Abbreviations

HPV-PCB: HPV Prevention and Control Board
HPV: Human papillomavirus
HCPs: Healthcare professionals
SWOT: Strengths, Weaknesses, Opportunities, and Threats
SARS-Cov-2: Several Acute Respiratory Syndrome Coronavirus 2
COVID-19: Coronavirus disease 2019
GDP: Gross Domestic Product
HAS: Haute Autorité de Santé
INCa: Institut National de Cancer
CTV: Comité Technique des Vaccinations
EMA: European Medicines Agency
MSM: Men who have sex with men
MenC: Meningococcus type C
MMR: Measles, Mumps, Rubella
GP: General Practitioners
FRANCIM: French Network of Cancer Registries
CMU: Coverture Médicale Universelle
US: United States
FDA: Food and Drug Administration
CRCDC: Cancer Screening Coordination Centres
GDPR: General Data Protection Regulation
QC: Quality Control
QA: Quality Assurance
LVSI: Lymphovascular space invasion
IVA: Acetic Acid Visual Inspection
ESGO: European Society of Gynecological Oncology
ESTRO: European Society of Radiotherapy & Oncology
ESP: European Society of Pathology
HNC: Head and neck cancer
OPC: Oropharyngeal cancer
GBS: Guillain-Barré Syndrome
